# The Antidiabetic and Antinephritic Activities of *Auricularia cornea* (An Albino Mutant Strain) via Modulation of Oxidative Stress in the db/db Mice

**DOI:** 10.3389/fimmu.2019.01039

**Published:** 2019-05-08

**Authors:** Di Wang, Xue Jiang, Shanshan Teng, Yaqin Zhang, Yang Liu, Xiao Li, Yu Li

**Affiliations:** ^1^Engineering Research Center of Chinese Ministry of Education for Edible and Medicinal Fungi, Jilin Agricultural University, Changchun, China; ^2^School of Life Sciences, Jilin University, Changchun, China

**Keywords:** *Auricularia cornea*, diabetes, diabetic nephropathy, oxidative stress, inflammation

## Abstract

This study first systematically analyzed the constituents of an albino mutant strain of *Auricularia cornea* (AU). After 8 weeks of continuous treatment with metformin (Met) (0.1 g/kg) and AU (0.1 and 0.4 g/kg), db/db mice showed hypoglycemic functioning, indicated by reduced bodyweight, food intake, plasma glucose, serum levels of glycated hemoglobin A1c and glucagon, hepatic levels of phosphoenolpyruvate carboxykinase and lucose-6-phosphatasem, and increased serum levels of insulin. The effect of hypolipidemic functions were indicated by suppressed levels of total cholesterol and triglyceride, and enhanced levels of hepatic glycogen and high-density lipoprotein cholesterol. The renal protective effect of AU was confirmed by the protection in renal structures and the regulation of potential indicators of nephropathy. The anti-oxidative and anti-inflammatory effects of AU were verified by a cytokine array combined with an enzyme-linked immunosorbent assay. AU decreased the expression of protein kinase C α and β2 and phosphor-nuclear factor-κB, and enhanced the expression of catalase, nuclear respiratory factor 2 (Nrf2), manganese superoxide dismutase 2, heme oxygenase-1 and−2, heat shock protein 27 (HSP27), HSP60, and HSP70 in the kidneys of db/db mice. The results confirmed that AU's anti-diabetic and anti-nephritic effects are related to its modulation on oxidative stress.

## Introduction

Diabetes mellitus (DM) is a progressive metabolic disease characterized by an imbalance in glucose homeostasis, impaired insulin secretion, and abnormal lipid and carbohydrate metabolism (1). The prevalence of DM has increased four-fold in the past three decades, and the global diabetic population is ~382 million (2).

Long-term hyperglycemia can induce secondary complications such as renal damage (3). Diabetic nephropathy (DN), a major cause of end-stage renal disease and cardiovascular disease (4), is characterized by an elevated lipid profile and increased oxidative stress (5). DN affects around 30% of patients with type 1 and 25% of patients with type 2 diabetes, which indicates excessive morbidity and mortality (4). Hyperglycemia induces the excessive production of mitochondrial superoxide and leads directly to the overproduction of reactive oxygen species (ROS), which can cause tubulointerstitial fibrosis and inflammatory cell infiltration (6). The accumulation of inflammatory cells in the glomerulus of DN patients stimulates the secretion of cytokines and chemokines, which transfer inflammatory cells to damaged areas (7).

ROS can be eliminated by nuclear respiratory factor 2 (Nrf2), a type of leucine zipper transcription factor that regulates the expression of phase 2 detoxification genes such as heme oxygenase 1 (HO-1) (8). Severe kidney damage has been observed in Nrf2 knockout diabetic mice (9). Nuclear factor-κB (NF-κB), an important inflammatory transcription factor, can be activated by extremely high levels of proinflammatory cytokines and consequently helps to generate more pro-inflammatory mediators under pathological conditions, including diabetes (10). NF-κB participates in the cellular response to stimulations, including ROS (11).

The conventional therapeutic agents for diabetes cannot effectively restore β-cell function, and the long-term nature of the treatment causes multiple side effects, including peripheral hyperinsulinemia, and hypoglycemia and increased cardiovascular risks (12, 13). These therapeutic strategies suppress blood glucose levels and reduce hypertension by blocking the renin-angiotensin system, which has a negative therapeutic effect on diabetic complications such as DN (5). Therefore, alternative agents with fewer side effects and greater activities against complications are needed. Edible fungi, which contain plenty of bioactive components with few adverse effects, are reported to show various pharmacological activities (14). Our previous studies confirmed that the antidiabetic activities of *Cordyceps militaris, Paecilomyces hepialid*, and *Inonotus obliquus* are related to the regulation of oxidative stress in diet-streptozotocin-induced diabetic Sprague-Dawley rats models (15–17). An albino mutant strain of *Auricularia cornea* entitled Yu Muer (AU) was first reported and cultured by the research team led by Prof. Li (the Chinese Academy of Engineering) at Jilin Agricultural University, Jilin, China. AU exhibits antineoplastic activity and antioxidant effects in H22 bearing mice (18). However, the antidiabetic and antinephritic activities of AU and their underlying mechanisms have not been reported.

The db/db mouse model exhibits insulin resistance at around 2 weeks of age and eventually develops hyperglycemia induced by β cell failure at 4–8 weeks, which accurately reflects the pathophysiology of diabetes (19). In the present study, the antidiabetic and antinephritic activities of AU and its possible oxidative stress-related mechanisms were analyzed on db/db mice.

## Materials and Methods

### Detection of AU Components

The cultured fruitbodies of AU (provided by Prof. Li's group at Jilin Agricultural University, Jilin, China) were shattered by a crusher and dry stored for the follow-up experiment. Figure S1 presents a picture of AU.

#### Main Components Analysis

The main components of AU, including total protein, total sugar, reducing sugar, crude fat, total ash, crude fiber, and total flavones, were assessed by the Kjeldahl method (20), phenol-sulfuric acid method (21), direct titration (22), Soxhlet extraction (23), combustion method (24), double differences method (25), and UV spectrophotometry (26), respectively. Total triterpenoids and mannitol were assessed by high performance liquid chromatography (HPLC) (27, 28).

#### Fatty Acids Analysis

AU was extracted with a 1:1 ratio of ether: petroleum ether (V:V) via evaporation at 80°C, then 0.5 M of NaOH in a methanol solution and 25% Boron trifluoride (BF3) were added stepwise and incubated at 60°C for 30 and 20 min, respectively. Finally, a saturated solution of NaCl and hexane was mixed with the samples, and the levels of fatty acids were analyzed using a gas chromatography-mass spectrometer (QP2010, Shimadzu, Japan) (29).

#### Amino Acids Analysis

AU was hydrolyzed by HCl (6 mol/L) at 110°C for 24 h, and the amino acid composition of AU was analyzed by HPLC using an Agilent 1260 (Agilent, California, America) equipped Agilent C18 column (4.6 × 250 mm × 5 μm) at 1.0 mL/min with mobile phase A (25 mM acetate buffer, pH 5.8) and mobile phase B (acetonitrile) (30).

#### Minerals Analysis

AU (0.5 g) was placed in a digestion tank and mixed with nitric acid (5 mL) to digest for 27 min (at 100, 140, 160, and 180°C, 3 min of each, and at 190°C for 15 min). The levels of minerals including zinc (Zn), kalium (K), ferrum (Fe), manganese (Mn), natrium (Na), cuprum (Cu), and calcium (Ca) were detected using inductively coupled plasma optical emission spectrometry (ICP-OES, optima 8,000) (31), and lead (Pb), selenium (Se), mercury (Hg), chromium (Cr), cadmium (Cd), and arsenic (As) were analyzed using inductively coupled plasma mass spectrometry (Thermo Fisher Scientific ICAPQ) (32).

### Animal Care and Experimental Design

The experimental animal protocol was approved by the Animal Ethics Committee of Jilin University (20170301). All procedures were carried out on the basis of the Laboratory Animal Care and Use recommendations, which are intended to reduce the use of animals and minimize animal distress. The male db/db mice and wild db/+ littermates in a C57BLKs/J background [8 weeks, SCXK (Su) 2015-0001] were purchased from the Nanjing Biomedical Research Institute of Nanjing University (Nanjing, China). Mice were housed at a temperature of 23 ± 1°C and humidity of 60% with a 12-h light-dark cycle (lights on 07:00–19:00) and free access to food and water. After 1 week of adaptation, the db/db mice with non-random blood glucose levels >11.1 mmol/L were considered to be diabetes. The mice were randomly divided into four groups (*n* = 12/group) and treated with 4.0 mL/kg of physiological saline (model group), Met at 0.1 g/kg (positive control group) and AU at doses of 0.1 and 0.4 g/kg (AU-treated groups) by gavage once per day, respectively, for eight consecutive weeks. The db/+ mice (*n* = 12) were orally treated with 4.0 mL/kg of physiological saline (control group) for eight consecutive weeks. AU fruiting body was pulverized using the ultrafine grinder (XDW-6A, Ji'nan Tatsu Micro Machinery Co. Ltd., Ji'nan, China) and mixed with physiological saline. Before administration, the mixture was shaken up. Throughout the experimental period, the body weights and blood glucose concentrations of the mice were measured once a week. Due to the limited amount of sera and tissues, we randomly stagger samples to guarantee a sample size of 10 for each of the following assays.

### Sample Collection and Parameter Determination

The mice were fasted for 2 h before sacrifice. Blood samples were collected and then centrifuged at 3,000 rpm for 10 min twice, and the collected sera were stored at −80°C. Tissues (kidney and liver) were harvested and washed in ice-cold physiological saline solution. Half of each tissue was homogenized in double distilled water and/or a radioimmunoprecipitation assay (RIPA) buffer (Sigma-Aldrich, USA) containing 1% protease inhibitor cocktail and 2% phenylmethanesulfonyl fluoride (Sigma-Aldrich, USA) and stored at −80°C for the subsequent experiment, while the other half was embedded with 10% neutral phosphate-buffered formalin for histopathological examination. Enzyme-linked immunosorbent assay (ELISA) commercial kits (Shanghai Yuanye Bio-Technology Co. Ltd., Shanghai, China) were used to determine the levels of granulocyte colony-stimulating factor (G-CSF, CK-E20002), glycated hemoglobin A1c (GhbA1c; CK-E20512), insulin (INS, CK-E20353), total cholesterol (TC; CK-E91839), triglyceride (TG; CK-E91733), high-density lipoprotein cholesterol (HDL-C; CK-E93031), alanine aminotransferase (ALT; CK-E90314), aspartate aminotransferase (AST; CK-E91386), and glucagon (GC; CK-E92275) in serum; the levels of interleukin (IL)-2 (CK-E20010), IL-1β (CK-E20533), IL-10 (CK-E20005), ROS (CK-E91516), microalbuminuria (MAU/ALB, CK-E95121), 6-keto-prostaglandin F1α (6-K-PGF1α; CK-E30144), and matrix metalloproteinase-9 (MMP-9; CK-E90157) in kidney; the level of N-acetyl-β-D-glucosidase (NAG; CK-E20276) in urine; the levels of phosphoenolpyruvate carboxykinase (PEPCK; CK-E93964) and glucose-6-phosphatase (G-6-Pase; CK-E94770) in liver; and the levels of glutathione peroxidase (GSH-Px; CK-E92669), superoxide dismutase (SOD; CK-E20348). and catalase (CAT; CK-E92636) in serum and kidney. Glycogen assay kits (A043) (Nanjing Jiancheng Bioengineering Institute, Nanjing, China) were used to detect the level of hepatic glycogen.

### Proteome Profiling of Kidney

Twenty-six cytokines from the kidney samples of db/db mice were quantified using a Cytokine Array Kit (ARY018, R&D Systems, Minneapolis, MN). Briefly, the kidney was excised and homogenized in RIPA buffer with 1% protease inhibitor cocktail (Sigma-Aldrich, USA). After centrifugation at 10,000 rpm for 10 min, the protein concentration of the supernatant was quantitated using a bicinchoninic acid (BCA) protein assay kit (Merck Millipore, USA). Membranes containing 26 different cytokine antibodies were blocked with BSA for 1 h at room temperature and then incubated with 100 μg of protein supernatant mixed with a cocktail of biotinylated detection antibodies. Streptavidin-HRP and chemiluminescence were used to detect the antibodies bound to the membrane antibodies. The membranes were then exposed and quantified using Image J software (National Institutes of Health, Bethesda, MD).

### Western Blot

One part of the kidney tissues obtained from the db/db mice was homogenized in RIPA buffer with a 1% protease inhibitor cocktail on ice for 30 min. After centrifugation (10,000 rpm for 10 min) and elimination of the precipitate, total protein concentrations were determined by BCA protein assay kit (Merck Millipore, USA). Denatured protein samples (40 μg) were subjected to 12% sodium dodecyl sulfate-polyacrylamide gel electrophoresis (SDS-PAGE) (Bio-Rad, USA) and then electro blotted onto 0.45 μm PVDF membranes (Bio Basic, Inc., USA). After blocking with 5% bovine serum albumin (BSA) for 4 h, the transferred membranes were incubated overnight at 4°C in the corresponding primary antibodies (at a dilution of 1:2,000) containing total-NF-κB (t-NF-κB, ab32536), phosphor-NF-κB (p-NF-κB, ab86299), Nrf2 (ab137550), catalase (CAT, ab16731), HO-1 (ab68477), HO-2 (ab90492), manganese superoxide dismutase 2 (SOD2, ab13533), protein kinase C alpha (PKC-α, ab23513), PKC β2 (ab32026), heat shock protein 27 (HSP27, ab12351), HSP 60 (ab45134), and HSP70 (ab181606) (Abcam, Cambridge, USA), and the reference protein glyceraldehyde-3-phosphate dehydrogenase (GAPDH; ABS16) (Merck Millipore, Darmstadt, Germany). The transferred membranes were washed five times with TBS buffer and then incubated with horseradish peroxidase-conjugated goat anti-rabbit secondary antibody (sc-3836) (Santa Cruz Biotechnology, Santa Cruz, USA) for 4 h at 4°C. The protein bands were established and fixed by Immobilon Western HRP substrate (Millipore Corporation, Billerica, USA). The relative intensity of protein expression was quantified using Image J software (National Institutes of Health, Bethesda, MD).

### Histopathological Observation

Ten percent formalin-fixed kidney tissues were dehydrated in ethyl alcohol (from 70 to 100%) and dealcoholized in xylene. Subsequently, the tissues were embedded in paraffin and cut into 5-mm thick sections. Sections were then deparaffinized in xylene and rehydrated in ethyl alcohol (from 100 to 70%) in reverse order. All specimens were stained with hematoxylin and eosin (H&E) and periodic acid Schiff (PAS) and assessed for kidney damage and inflammation under an inverted microscope CKX41 (Olympus, Japan).

### Statistical Analysis

All data were expressed as the mean ± S.E.M. Differences were determined by one-way analysis of variance followed by *post-hoc* multiple comparisons (Dunn's test) using SPSS 16.0 software (IBM Corporation, Armonk, USA). Statistical significance was declared for *p*-values under 0.05.

## Results

### Composition of AU

The AU consisted of 56.9% total sugar, 2.8% reducing sugar, 8.1% protein, 4.2% total ash, 2.4% crude fat, 8.0% crude fiber and 3.1 × 10^−4^% total triterpenoids (Table 1). Among 17 types of amino acid detected, the concentrations of glutamic acid, aspartic acid, leucine and arginine were higher than others (Table 1). Seven minerals, Zn, Fe, Mn, Ca, Cu, Na, and K were detected in AU (Table 1).

**Table 1:** Main components of AU.

	**Compounds**	**Contents (%)**	**Compounds**	**Contents (%)**	**Compounds**	**Contents (%)**
Main components	Total protein	8.1	Total sugar	56.9	Reducing sugar	2.8
	Crude fat	2.4	Total ash	4.2	Crude fiber	8.0
	Total triterpenoids (×10^−4^)	3.1	Total flavones	ND^I^	Mannitol	ND^II^
Amino acid	Aspartic acid (Asp)	0.6	Glutamic acid (Glu)	0.7	Cystine (Cys)	0.3
	Serine (Ser)	0.4	Glycine (Gly)	0.3	Histidine (His)	0.2
	Arginine (Arg)	0.5	L-Threonine (Thr)	0.3	Alanine (Ala)	0.4
	Proline (Pro)	0.3	Tyrosine (Tyr)	0.2	Valine (Val)	0.3
	DL-Methionine (Met) (×10^−2^)	6.0	Isoleucine (Ile)	0.2	Leucine (Leu)	0.5
	Phenylalanine (Phe)	0.4	Lysine (Lys)	0.3		
Minerals	Zinc (Zn) (×10^−3^)	3.6	Ferrum (Fe) (×10^−3^)	4.3	Manganese (Mn) (×10^−3^)	0.5
	Calcium (Ca)	0.1	Cuprum (Cu) (×10^−3^)	0.6	Natrium (Na) (×10^−2^)	1.7
	Kalium (K)	1.1	Lead (Pb) (×10^−5^)	1.2	Mercury (Hg)	ND^III^
	Chromium (Cr) (×10^−4^)	5.0	Arsenic (As) (×10^−6^)	4.0	Cadmium (Cd)	ND^III^
	Selenium (Se) (×10^−6^)	2.6				

*AU, Auricularianigricans; ND, not detected; ND^I^, the detection limit was 1 g/kg; ND^II^, the detection limit was 0.056 g/kg; ND^III^, the detection limit was 20 μg/kg*.

The concentrations of Pb, Cr, As, and Se in the AU were below the limit of detection, and the AU didn't contain Hg or Cd (Table 1). Among 35 types of fatty acid tested, only 16 types of fatty acid were existed in the AU (Table 2).

**Table 2:** The composition and percentage content of fatty acids.

**Compounds**	**Contents (%)**	**Compounds**	**Contents (%)**	**Compounds**	**Contents (%)**
Octoic acid (C8:0)	ND^I^	Heptadecenoic acid (C17:1) (×10^−3^)	4.0	Docosanoic acid (C22:0) (×10^−2^)	2.2
Capric acid (C10:0)	ND^II^	Stearic acid (C18:0)	0.2	Eicosatrienoic acid (C20:3n6)	ND^XI^
Undecanoic acid (C11:0)	ND^III^	Trans-oleic acid (C18:1n9t) (×10^−3^)	2.0	Erucic acid (C22:1n9)	ND^XII^
Lauric acid (C12:0)	ND^IV^	Oleic acid (C18:1n9c)	0.6	Eicosatrienoic acid (C20:3n3)	ND^XIII^
Tridecanoic acid (C13:0)	ND^V^	Trans-linoleic acid (C18:2n6t)	ND^VII^	Arachidonic acid (C20:4n6)	ND^XIV^
Myristic acid (C14:0) (×10^−3^)	4.0	Linoleic acid (C18:2n6c)	0.8	Tricosanoic acid (C23:0) (×10^−3^)	4.0
Myristoleic acid (C14:1)	ND^VI^	Arachidic acid (C20:0) (×10^−2^)	1.3	Docosadienoic acid (C22:2n6)	ND^XV^
Pentadecanoic acid (C15:0) (×10^−2^)	3.4	γ-linolenic acid (C18:3n6)	ND^VIII^	Eicosapentaenoic acid (C20:5n3)	ND^XVI^
Pentadecenoic acid (C15:1)	ND^VII^	Eicosaenoic acid (C20:1n9) (×10^−2^)	2.8	Tetracosanoic acid (C24:0) (×10^−2^)	3.8
Hexadecanoic acid (C16:0)	0.3	α-linolenic acid (C18:3n3)	ND^IX^	Nervonic acid (C24:1n9)	ND^XVII^
Palmitoleic acid (C16:1) (×10^−3^)	4.0	Heneicosanoic acid (C21:0)	ND^X^	Docosahexaenoic acid (C22:6n3)	ND^XVIII^
Heptadecanoic acid (C17:0) (×10^−3^)	8.0	Eicosadienoic acid (C20:2) (×10^−3^)	3.0		

*ND, not detected; ND^I^, the detection limit was 4.20 mg/kg; ND^II^, the detection limit was 3.83 mg/kg; ND^III^, the detection limit was 3.54 mg/kg; ND^IV^, the detection limit was 2.99 mg/kg; ND^V^, the detection limit was 2.91 mg/kg; ND^VI^, the detection limit was 2.82 mg/kg; ND^VII^, the detection limit was 2.64 mg/kg; ND^VIII^, the detection limit was 2.51 mg/kg; ND^IX^, the detection limit was 2.36 mg/kg; ND^X^, the detection limit was 2.05 mg/kg; ND^XI^, the detection limit was 2.68 mg/kg; ND^XII^, the detection limit was 2.42 mg/kg; ND^XIII^, the detection limit was 3.21 mg/kg; ND^XIV^, the detection limit was 4.66 mg/kg; ND^XV^, the detection limit was 2.88 mg/kg; ND^XVI^, the detection limit was 3.31 mg/kg; ND^XVII^, the detection limit was 4.83 mg/kg; ND^XVIII^, the detection limit was 4.33 mg/kg*.

### The Hypoglycemic Effect of AU on db/db Mice

Compared with db/+ mice, the db/db mice showed increased bodyweight and changes in the organ indices of the kidney, spleen and liver (*P* < 0.001, Table 3). After 8 weeks of administration of AU at doses of 0.1 and 0.4 g/kg, bodyweight was reduced by 9.1 and 13.6%, respectively (*P* < 0.05, Table 3). AU at 0.4 g/kg strongly enhanced the kidney and spleen indices and reduced the liver indices (*P* < 0.05, Table 3). The high levels of food intake observed in the db/db mice were also suppressed by Met and AU after 8 weeks of administration (*P* < 0.05; Table S1).

**Table 3:** The effects of AU on body weights and organ indices.

	**Week**	**db/+**	**db/db**	**0.1 g/kg Met**	**0.1 g/kg AU**	**0.4 g/kg AU**
Body weights (g)	1	20.0 ± 0.3	43.0 ± 0.5^###^	43.1 ± 0.8	43.9 ± 0.4	44.3 ± 0.8
	2	21.1 ± 0.3	45.1 ± 0.7^###^	44.9 ± 0.7	44.7 ± 0.5	44.8 ± 1.0
	3	21.0 ± 0.3	45.3 ± 0.7^###^	44.6 ± 1.0	43.8 ± 0.9	44.7 ± 1.2
	4	20.9 ± 0.4	45.5 ± 1.0^###^	44.1 ± 0.8	45.4 ± 0.9	45.4 ± 1.1
	5	21.0 ± 0.3	47.7 ± 0.9^###^	44.0 ± 0.8^*^	46.1 ± 0.9	45.9 ± 1.1
	6	21.8 ± 0.3	52.4 ± 0.9^###^	46.6 ± 0.9^**^	50.6 ± 0.7	47.1 ± 1.4^*^
	7	21.6 ± 0.3	53.6 ± 0.6^###^	48.9 ± 1.2^*^	49.0 ± 1.0^*^	48.4 ± 1.5^*^
	8	22.0 ± 0.3	54.7 ± 0.6^###^	50.1 ± 1.1^**^	49.5 ± 1.2^*^	49.0 ± 1.7^*^
	9	21.9 ± 0.6	55.9 ± 0.5^###^	52.6 ± 0.3^**^	50.8 ± 1.4^*^	48.3 ± 2.1^*^
Organ indices (%)	Kidney	1.28 ± 0.03	0.69 ± 0.02^###^	0.78 ± 0.03^*^	0.80 ± 0.07	0.85 ± 0.05^*^
	Spleen	0.34 ± 0.06	0.14 ± 0.03^###^	0.14 ± 0.01	0.21 ± 0.06^**^	0.20 ± 0.08^*^
	Liver	4.04 ± 0.12	6.78 ± 0.14^###^	6.66 ± 0.13	6.52 ± 0.19	6.11 ± 0.11^*^

*The data were analyzed using a one-way ANOVA and they are expressed as means ± S.E.M. (n = 10)*.

### P < 0.001 vs. db/+ mice;

* P < 0.05, and

** *P < 0.01 vs. non-treated db/db mice*.

**Figure 1:**
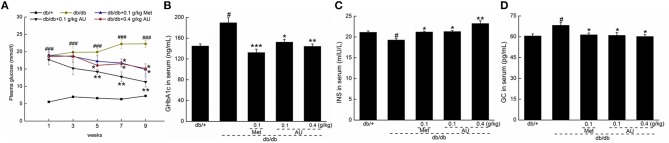
The hypoglycemic effect of AU on db/db mice. **(A)** AU reduced the fasting plasma glucose in db/db mice. AU regulated the serum levels of **(B)** GHbA1c, **(C)** INS, and **(D)** GC in db/db mice after 8-week administration. The data were expressed as means ± S.E.M. (*n* = 10) and analyzed using a one-way ANOVA. # *P* < 0.05 vs. db/+ mice, ### *P* < 0.001 vs. db/+ mice, ^*^P < 0.05, ^**^P < 0.01 and ^***^P < 0.001 vs. non-treated db/db mice. GHbA1c, glycated hemoglobin A1c; INS, insulin; GC, glucagon.

Increased blood glucose levels were observed in the db/db mice compared with the db/+ mice. Similar to Meet, AU remitted the increased levels of blood glucose (*P* < 0.05, Figure 1A). The db/db mice showed significantly elevated levels of GHbA1c and GC and diminished levels of INS in serum, all of which were reversed after administration with Met and AU (*P* < 0.05, Figures 1B–D).

### The Hypolipidemic and Liver Protective Effects of AU on db/db Mice

**Figure 2:**
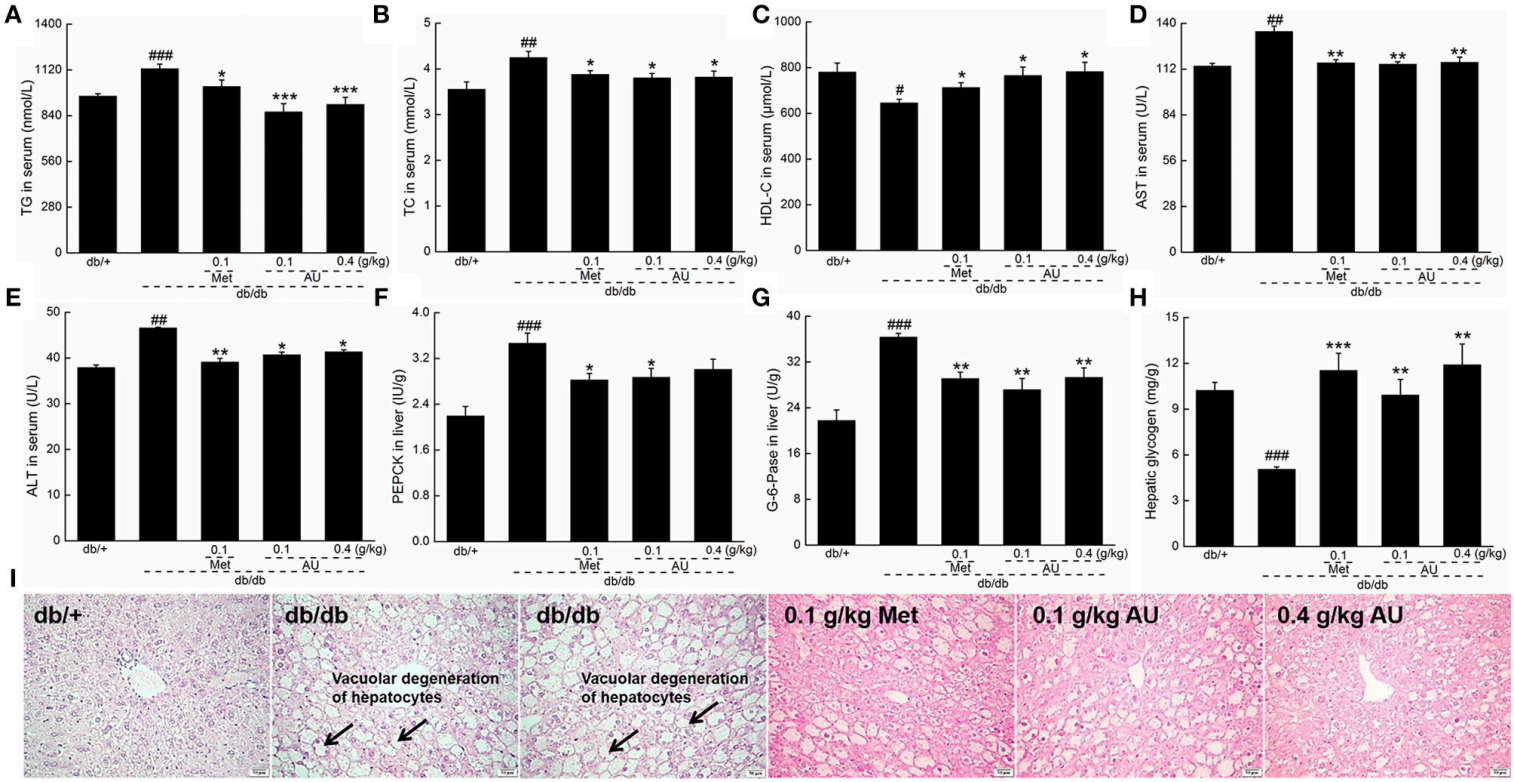
The hypolipidemic and liver protective effects of AU on db/db mice. AU regulated the serum levels of **(A)** TG, **(B)** TC, **(C)** HDL-C, **(D)** AST, and **(E)** ALT in db/db mice. AU reduced the levels of **(F)** PEPCK and **(G)** G-6-Pase in liver and enhanced the levels of **(H)** hepatic glycogen in db/db mice. The data were expressed as means ± S.E.M. (*n* = 10) and analyzed using a one-way ANOVA. ^#^*P* < 0.05, ^##^*P* < 0.01, and ^###^*P* < 0.001 vs. db/+ mice, ^*^P < 0.05, ^**^P < 0.01, and ^***^P < 0.001 vs. non-treated db/db mice. **(I)** Histopathological analysis in liver via H&E staining (scale bar: 50 μm; magnification: 200×). TG, triglyceride; TC, total cholesterol; HDL-C, high-density lipoprotein cholesterol; AST, aspartate aminotransferase; ALT, alanine aminotransferase; PEPCK, phosphoenolpyruvate carboxykinase; G-6-Pase, glucose-6-phosphatase; H&E, hematoxylin eosin.

Hyperlipoproteinemia is a common complication of DM (33). Compared with vehicle-treated db/db mice, serum TG and TC levels were significantly decreased (*P* < 0.05, Figures 2A,B); while the serum HDL-C concentration was increased (*P* < 0.05, Figure 2C) after 8 weeks of AU treatment.

The liver plays a significant role in blood glucose homeostasis, lipid metabolism, and glucose storage (34). ALT and AST, which remain at high levels in diabetes patients, reflect the impaired liver function (35). Similar to Meet, the 8-week AU treatment resulted in 11.2 and 14.0% reductions in the serum levels of ALT and AST, respectively (*P* < 0.05, Figures 2D,E).

Gluconeogenesis is one of the major pathways for the production of endogenous glucose. PEPCK and G-6-Pase are two rate-limiting enzymes that regulate hepatic gluconeogenesis (36). Compared with the db/+ mice, enhanced levels of PEPCK, and G-6-Pase (*P* < 0.001, Figures 2F,G) were observed in the db/db mice, which were suppressed by Met and AU (*P* < 0.05, Figures 2F,G). AU enhanced hepatic glycogen levels by >94.1% in db/db mice (*P* < 0.01, Figure 2H). AU and Met improved vacuolar degeneration of hepatocytes in the db/db mice in pathological examinations, further confirming their hepatoprotective effects (Figure 2I).

### The Renal Protection of AU on db/db Mice

**Figure 3:**
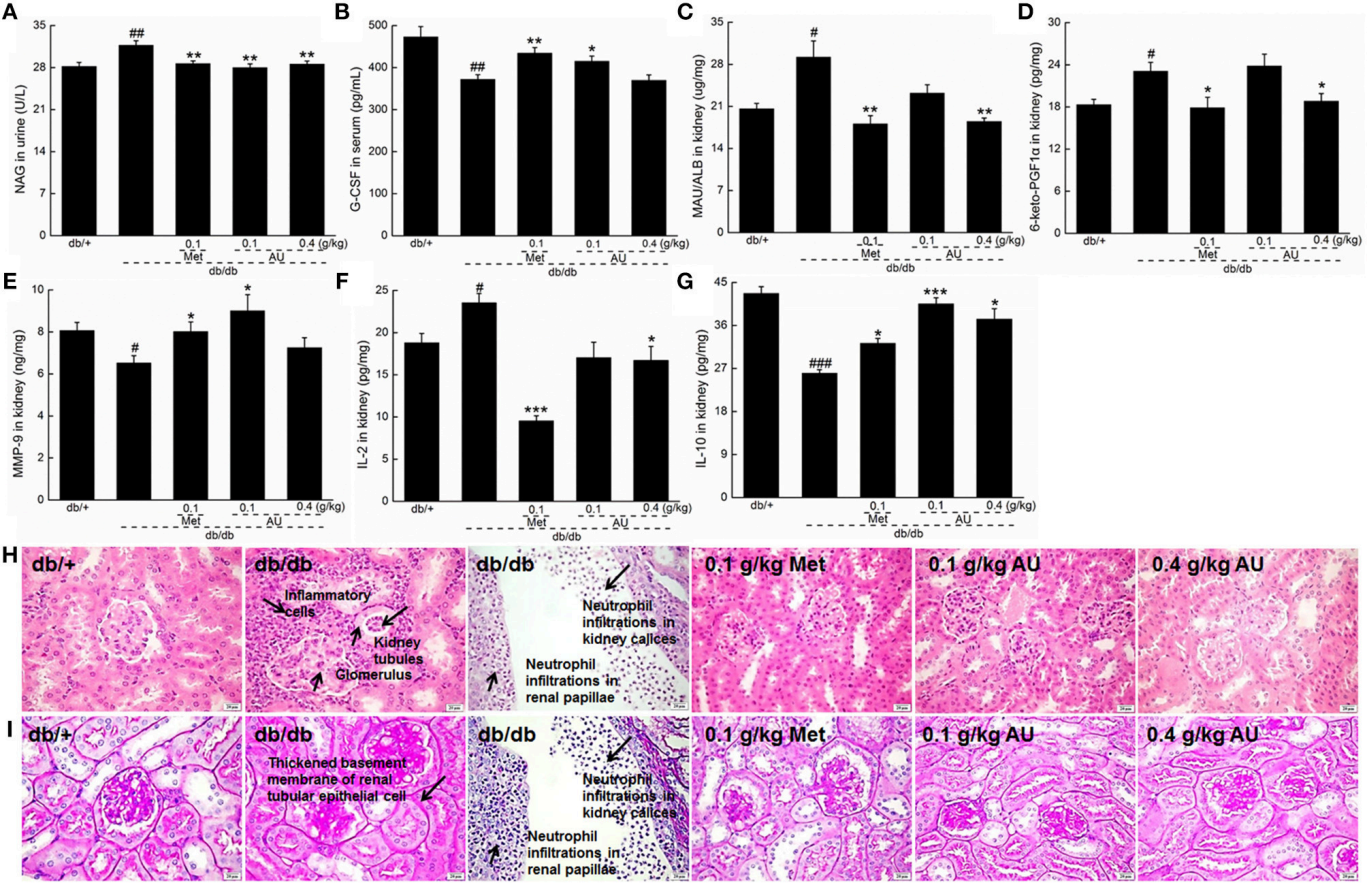
The renal protection of AU via anti-inflammation on db/db mice. **(A)** AU reduced the NAG levels in urine of db/db mice. **(B)** AU enhanced the serum levels of G-CSF in db/db mice. AU reduced the levels of **(C)** MAU/ALB and **(D)** 6-keto-PGF1α and enhanced the levels of **(E)** MMP-9 in kidney of db/db mice. AU reduced the levels of **(F)** IL-2 and enhanced the levels of **(G)** IL-10 in kidney of db/db mice. The data were analyzed using a one-way ANOVA and they are expressed as means ± S.E.M. (*n* = 10). ^#^*P* < 0.05, and ^##^*P* < 0.01 vs. db/+ mice, ^###^ *P* < 0.001 vs. db/+ mice, ^*^P < 0.05, ^**^P < 0.01, and ^***^P < 0.001 vs. non-treated db/db mice. Histopathological analysis in kidney via **(H)** H&E staining (scale bar: 20 μm; magnification: 400×) and **(I)** PAS staining (scale bar: 20 μm; magnification: 400×). NAG, N-acetyl-β-D-glucosidase; G-CSF, granulocyte colony-stimulating factor; MAU/ALB, microalbuminuria; 6-keto-PGF1α, 6-keto-prostaglandin F1α; MMP-9, matrix metalloproteinase-9; IL, interleukin; H&E, hematoxylin eosin; PAS, periodic acid Schiff.

As a specific and sensitive index of renal tubular damage (37), the high levels of NAG in urine were significantly reduced by AU in the db/db mice (*P* < 0.01; Figure 3A). Furthermore, 8 weeks of AU administration resulted in an 11.5% increment in serum levels of G-CSF (*P* < 0.05, Figure 3B), a 36.6% reduction in renal levels of MAU/ALB (*P* < 0.01, Figure 3C), and a 21.2% reduction in renal levels of 6-keto-PGF1α (*P* < 0.05, Figure 3D). Meanwhile, AU at 0.1 g/kg enhanced the renal levels of MMP-9 by 38.5% (*P* < 0.05, Figure 3E) in the db/db mice.

The tubulointerstitial and glomerular damage caused by DN is closely related to inflammatory cytokines (12). Among the detected inflammatory cytokines, 8 weeks of AU administration resulted in a reduction of >28.9% in IL-2 levels and an increment of >24.2% in IL-10 levels in the kidney of the db/db mice (*P* < 0.05, Figures 3F,G).

The renal protective effect of AU was further confirmed by the H&E and PAS staining. The neutrophil infiltrations in renal papillae, inflammatory cell infiltrations, and thickened basement membrane of renal tubular epithelial cells in the db/db mice were all improved by 8 weeks of AU and Met administration (Figures 3H,I). Encouragingly, AU had no effect on the organ structures of the spleen, indicating its safe use in animals (Figure S2).

### Antioxidative Effects of AU on db/db Mice

The cytokines-related to inflammation and oxidative stress in the kidneys of db/db mice treated with AU were systematically screened using high-throughput renal antibody chip analysis. Among the 26 detected cytokines, AU influenced the levels of HSP27, HSP60, HSP70, and SOD2 oxidative stress-related cytokines in the kidney (Figure S3 and Table S2). The overproduction of superoxide induced by hyperglycemia leads to cellular damage, which can be equilibrated by the activities of antioxidant and redox factors (38). Based on the results of the cytokine array assay and ELISA detection, the underproduction of CAT, GSH-Px, and SOD were noted in the kidneys of the db/db mice (*P* < 0.05, Table 4), all of which were significantly enhanced by Met and AU administration (*P* < 0.05, Table 4). Eight weeks of AU administration resulted in a >26.6% reduction in ROS levels in the kidneys of the db/db mice (*P* < 0.05, Table 4). AU also enhanced the serum levels of CAT, GSH-Px, and SOD in the db/db mice (*P* < 0.05, Figure S4).

**Table 4:** The effects of AU on oxidative stress related factors in kidney of mice.

	**db/+**	**db/db**	**0.1 g/kg Met**	**0.1 g/kg AU**	**0.4 g/kg AU**
CAT (U/mg)	9.0 ± 0.5	7.2 ± 0.3^#^	8.7 ± 0.6^*^	10.0 ± 1.1^*^	8.3 ± 0.4^*^
GSH-Px (U/mg)	74.3 ± 6.6	44.1 ± 2.1^###^	53.1 ± 3.9^*^	65.1 ± 6.3^**^	58.5 ± 5.3^*^
SOD (U/mg)	37.0 ± 2.6	25.3 ± 2.1^##^	32.9 ± 2.7^*^	35.5 ± 3.3^*^	34.2 ± 3.4^*^
ROS (U/mg)	48.6 ± 0.3	66.1 ± 4.9^#^	41.0 ± 1.8^***^	48.5 ± 4.8^*^	44.8 ± 2.3^**^

*The data were analyzed using a one-way ANOVA and they are expressed as means ± S.E.M. (n = 10)*.

# *P < 0.05*,

## P < 0.01, and

### P < 0.001 vs. db/+ mice;

* *P < 0.05*,

** P < 0.01, and

*** *P < 0.001 vs. non-treated db/db mice. CAT, catalase; GSH-Px, glutathione peroxidase; SOD, superoxide dismutase; ROS, reactive oxygen species*.

**Figure 4:**
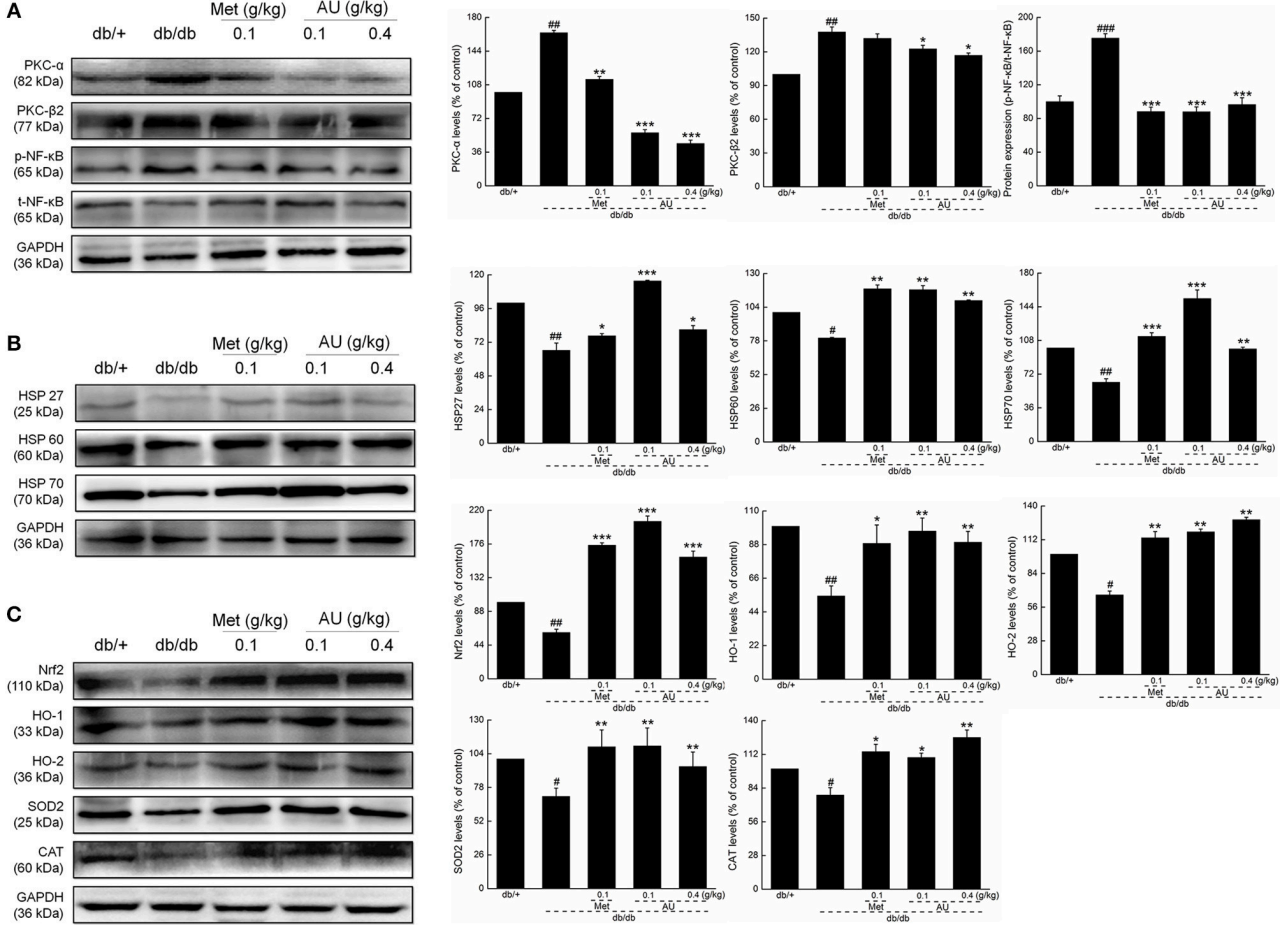
The anti-oxidation and anti-inflammation of AU may be related to Nrf2 signaling. **(A)** AU reduced the expression levels of PKC-α, PKC β2, and p-NF-κB in the kidney of db/db mice. **(B)** AU enhanced the expression levels of HSP26, HSP60, and HSP70 in the kidney of db/db mice. **(C)** AU enhanced the expression levels of Nrf2, HO-1, HO-2, SOD2, and CAT in the kidney of db/db mice. The data on quantified protein expression were normalized to the levels of GAPDH, except for p-NF-κB, which was normalized to the expression levels of t-NF-κB. The data were analyzed using a one-way ANOVA and they are expressed as means ± S.E.M. (*n* = 10). ^#^*P* < 0.05, ^##^*P* < 0.01, and ^###^*P* < 0.001 vs. db/+ mice, ^*^P < 0.05, ^**^P < 0.01, and ^***^P < 0.001 vs. non-treated db/db mice. PKC, protein kinase C; NF-κB, nuclear factor-κB; HSP, heat shock protein; Nrf2, nuclear respiratory factor 2; HO-1, heme oxygenase 1; HO-2, heme oxygenase 2; SOD2, manganese superoxide dismutase 2; CAT, catalase; GAPDH, glyceraldehyde-3-phosphate dehydrogenase.

Based on the results of the high-throughput renal antibody chip analysis, we further studied the effects of AU on oxidative stress. Compared with the model mice, the expressions levels of PKC-α, PKC-β2, and p-NF-κB in kidney tissues were significantly downregulated by AU (*P* < 0.05, Figure 4A). Met and AU administration increased the expression levels of HSP27, HSP60, and HSP70 by western blot, as a validation of the high-throughput renal antibody chip analysis (*P* < 0.05, Figure 4B). The expressions levels of Nrf2, HO-1, HO-2, SOD2, and CAT were significantly upregulated in the kidneys of the db/db mice after 8 weeks of AU administration, indicating its antioxidant activities (*P* < 0.05, Figure 4C).

## Discussion

AU contains multifarious nutritive materials (7 varieties of mineral, 17 varieties of amino acid, and 16 varieties of fatty acid). Polysaccharides extracted from fungi show various pharmacological activities, including antidiabetic properties (17). Selenium, an essential trace element, helps to prevent diabetes effectively via antioxidation (14). Based on the contents of AU and our experimental data, we successfully confirmed that AU exhibited a hypoglycemic effect by reducing blood glucose levels, modulating glucose tolerance, and recovering the serum levels of GHbA1c, GC, and INS. The high level of food intake observed in the db/db mice was strongly reversed by Met and AU. AU appeared to affect glucose metabolism mainly by reducing body weight and altering appetite. Among patients with diabetes, 55–80% have glycogen deposition abnormalities and steatohepatitis in their livers. Patients also showed a decreased entry rate of glucose into peripheral tissues, elevated hepatic glucose production, and gluconeogenesis (39). The glucose disposal and glycogen accumulation stimulated by insulin is an important way of regulating glucose concentration (34). Glycogen, a primary intracellular storable form of glucose, can be produced by gluconeogenesis. PEPCK and G-6-Pase are two rate-limiting enzymes that regulate hepatic gluconeogenesis and accelerate the transformation of glycogen, fat, and protein into glucose. The inhibition of PEPCK and G-6-Pase expression can effectively regulate the increased blood glucose (36, 40), which is consistent with the results of our present study. Lipid peroxidation caused by hyperglycemia induces liver damage in diabetes (41). Insufficient insulin leads to the accumulation of lipids, specifically TG and TC, in hyperglycemic patients, and thus causes diabetes-related complications (42). The accumulation of excessive adipose cells in the liver leads to hepatic steatosis and further fatty liver damage (43). The beneficial effects of AU on lipid metabolism indices and liver structures strongly confirmed its protective effect on the liver in the db/db mice. Combined with hyperglycemia and insulin resistance during the development of DN, oxidative stress, and inflammation are reported to be involved in inducing tubular fibrosis and mesangial expansion (44). The renal protective effect of AU was demonstrated by the down-regulation of the levels of NAG in urine and MAU/ALB in the kidney, and up-regulation of the levels of G-CSF and MMP-9 in the kidney of the db/db mice. Microalbuminuria is an early predictive risk factor for nephropathy, which can lead to abnormalities in the kidney tissues such as nodules and expansions of the mesangium (45). The fibrinolytic activity of MMP-9 plays a beneficial role in preventing crescentic proliferative glomerulonephritis in mice (46). G-CSF reduces pro-inflammatory cytokine expression and prevents the endothelialization of damaged vascular tissue (47), which helps to stop the progression of DN in rats (48).

Intense inflammatory reaction is accompanied by the progression of DN, which can develop into nephritides in the late stage (49). AU increased the levels of IL-2 and IL-10 in the kidneys of db/db mice, further demonstrating its renoprotective effect. Proinflammatory CD4+ cells are activated by the overproduction of IL-2, leading to the deterioration of glomerular damage by recruiting neutrophils (50). As an efficient anti-inflammatory cytokine, IL-10 can improve hyperglycemia, and insulin resistance (51). NF-κB signaling is exacerbated in the glomeruli and renal tubes in patients with DN, and regulates the expression of inflammatory mediators and proinflammatory cytokines (10, 52). Hyperglycemia in diabetes leads to the activation of PKC, which further enhances the activation of NF-κB (53). The renal protection of AU in db/db mice may be partially related to its anti-inflammatory effects via the regulation of NF-κB signaling.

Hyperglycemia and lipotoxicity, a state induced by dyslipidemia, lead to renal injury due to oxidative stress through the production of excess ROS (54). Oxidative stress triggers inflammatory reactions, such as basement membrane thickening and inflammatory cell infiltration, by activating NF-κB signaling, and finally exacerbates kidney damage in DN (49, 55). On the one hand, heat shock proteins (HSPs) contribute to protein homeostasis, accelerate regeneration, and minimize injury, thus protecting cells against various stressors such as oxidative stress as part of the defense system (56). Alternatively, oxidative damage can be prevented by enhancing the activities of antioxidant enzymes including CAT, GSH-Px, and SOD, which strengthen the response of the antioxidant defense system (57). SOD catalyzes the translation of superoxide radicals into hydrogen peroxide, which is then decomposed into oxygen and water by CAT, thus preventing the accumulation of ROS (58). AU successfully regulated Nrf2 and its downstream targets. Nrf2 can neutralize ROS by activating and regulating intracellular antioxidant effects (59). Preventing the degradation of Nrf2 resulted in the transcription of downstream antioxidant enzymes such as HO-1 and SOD (60). Evidence suggests that Nrf2-dependent ARE activation influences the upregulation of HSPs such as HSP70 (61). The antidiabetic and antinephritic activities of AU in db/db mice may be partially related to its anti-oxidative and anti-inflammatory activities via Nrf2 signaling. However, more experiments need to be performed to prove Nrf2 is a downstream effector of AU to perform antioxidative effects.

There were some limitations to our present study. High-throughput renal antibody chip analysis shows that AU influences the levels of apoptosis-related cytokines in kidneys, which we failed to detect in the present study. We will further investigate the anti-apoptotic effects of AU as part of its renal protection effect in db/db mice. Although we detected the main components of the albino mutant strain of *A. cornea*, based on the present results, we are still hard to conclude which compounds cause the antidiabetic and antinephritic activities. In our ongoing experiments, we have already separated the polysaccharides from the albino mutant strain of *A. cornea*, which showed hyperglycemic effects in db/db mice. We will further study whether the polysaccharides are responsible for these effects in AU. Furthermore, we only proved that AU attenuated diabetes and its following kidney oxidative pressure and inflammation. However, AU's pharmacological effect at macroalbuminuria stage still needs further investigation.

In conclusion, the anti-diabetic and anti- nephritic effects of AU and its possible anti-oxidation and anti-inflammation mechanisms-possible related to Nrf2 signaling- were explored in db/db diabetic models.

## Ethics Statement

The experimental animal protocol was approved by the Animal Ethics Committee of Jilin University (20170301). All efforts were carried out on the basis of the recommendations of Laboratory Animal Care and Use, which were made to reduce the use of animals and minimize animal distress. The male db/db mice and wild db/+ littermates in a C57BLKs/J background [8 weeks, SCXK (Su) 2015-0001] were purchased from the Nanjing Biomedical Research Institute of Nanjing University (Nanjing, China). Animals were housed at the temperature of 23 ± 1°C and humidity of 60% with a 12-h light-dark cycle (lights on 07:00–19:00) and free access to food and water.

## Author Contributions

XL and YuL: conceptualization. XJ, ST, YZ, and YaL: experiment and result. DW and XJ: article writing. All authors listed have made a substantial, direct and intellectual contribution to the work, and approved it for publication.

### Conflict of Interest Statement

The authors declare that the research was conducted in the absence of any commercial or financial relationships that could be construed as a potential conflict of interest.
