# Supplementary material for: The Antidiabetic and Antinephritic Activities of Auricularia cornea (An Albino Mutant Strain) via Modulation of Oxidative Stress in the db/db Mice
Source: Front Immunol. 2019 May 8;10:1039. doi: 10.3389/fimmu.2019.01039 (PMC6517500; doi:10.3389/fimmu.2019.01039)
Supplement: Supplementary file 1 [file Data_Sheet_1.doc]

**Table S1. The effect of AU on food intake of db/db mice**

|  | db/+ | db/db | 0.1 g/kg Met | 0.1 g/kg AU | 0.4 g/kg AU |
| --- | --- | --- | --- | --- | --- |
| Food intake (g/10g) | 1.27±0.09 | 2.37±0.23^##^ | 1.54±0.09^*^ | 1.44±0.10^**^ | 1.52±0.09^*^ |

The data were analyzed using a one-way ANOVA and they are expressed as means ± S.E.M. (n = 10). ^##^ *P* < 0.01 *versus* db/+ mice; * *P* < 0.05 and ** *P* < 0.01 *versus* non-treated db/db mice.

| Coordinate | Target | Fold (*vs.* db/+) | | |  |
| --- | --- | --- | --- | --- | --- |
|  |  | db/db | db/db+Met | db/db+AU | |
| B 3; B 4 | ADAMTS1 | 0.88 | 1.14 | 1.25 | |
| B 5; B 6 | Bcl-2 | 0.83 | 1.16 | 0.97 | |
| B 7; B 8 | Carbonic Anhydrase IX | 1.09 | 1.66 | 1.19 | |
| B 9; B 10 | Cited-2 | 1.02 | 1.55 | 1.22 | |
| B 11; B 12 | COX-2 | 1.29 | 1.62 | 1.25 | |
| B 13; B 14 | Cytochrome c | 1.32 | 1.76 | 1.45 | |
| B 15; B 16 | Dkk-4 | 0.98 | 1.60 | 1.30 | |
| B 17; B 18 | FABP-1 | 1.06 | 1.50 | 1.34 | |
| **B 19; B 20** | **HIF-1alpha** | **1.00** | **1.38** | **1.33** | |
| **C 3; C 4** | **HIF-2alpha** | **0.82** | **1.28** | **1.19** | |
| **C 5; C 6** | **Phospho-HSP27 (S78/S82)** | **0.80** | **1.29** | **1.01** | |
| **C 7; C 8** | **HSP60** | **0.98** | **1.76** | **1.17** | |
| **C 9; C 10** | **HSP70** | **0.92** | **1.29** | **0.86** | |
| C 11; C 12 | IDO | 1.07 | 1.61 | 1.36 | |
| C 13; C 14 | Phospho-JNK Pan (T183/Y185) | 0.95 | 1.51 | 1.23 | |
| **C 15; D 16** | **NFkappaB1** | **1.04** | **1.44** | **1.22** | |
| C 17; C 18 | p21/CIP1 | 0.85 | 1.21 | 1.01 | |
| C 19; C 20 | p27 | 0.97 | 1.26 | 1.19 | |
| D 3; D 4 | Phospho-p38alpha (T180/Y182) | 0.84 | 1.26 | 1.12 | |
| D 5; D 6 | Phospho-p53 (S46) | 0.78 | 1.27 | 0.81 | |
| D 7; D 8 | PON1 | 0.80 | 1.95 | 0.91 | |
| D 9; D 10 | PON2 | 0.99 | 1.53 | 0.98 | |
| D 11; D 12 | PON3 | 1.05 | 1.66 | 1.29 | |
| D 13; D 14 | Thioredoxin-1 | 1.13 | 1.53 | 1.32 | |
| D 15; D 16 | SIRT2 | 0.91 | 1.44 | 1.38 | |
| **D 17; D 18** | **SOD2** | **1.12** | **1.53** | **1.28** | |

**Table S2. The regulatory effects of AU on cytokines in kidney of db/db mice**

**
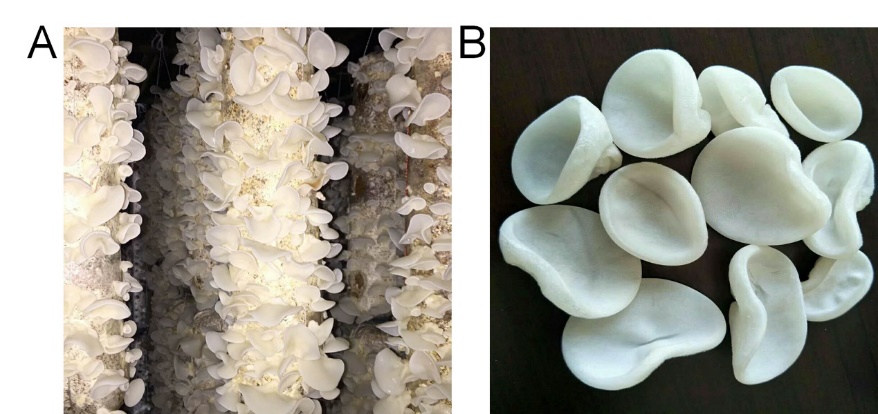
**

**Fig.S1.** The picture of *A. cornea* (an albino mutant strain).

**
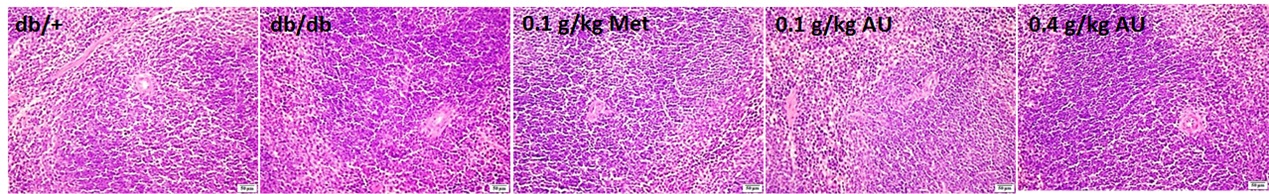
**

**Fig.S2.** Histopathological analysis in spleen was shown by hematoxylin & eosin staining (scale bar: 50 μm; magnification: 200×).

**
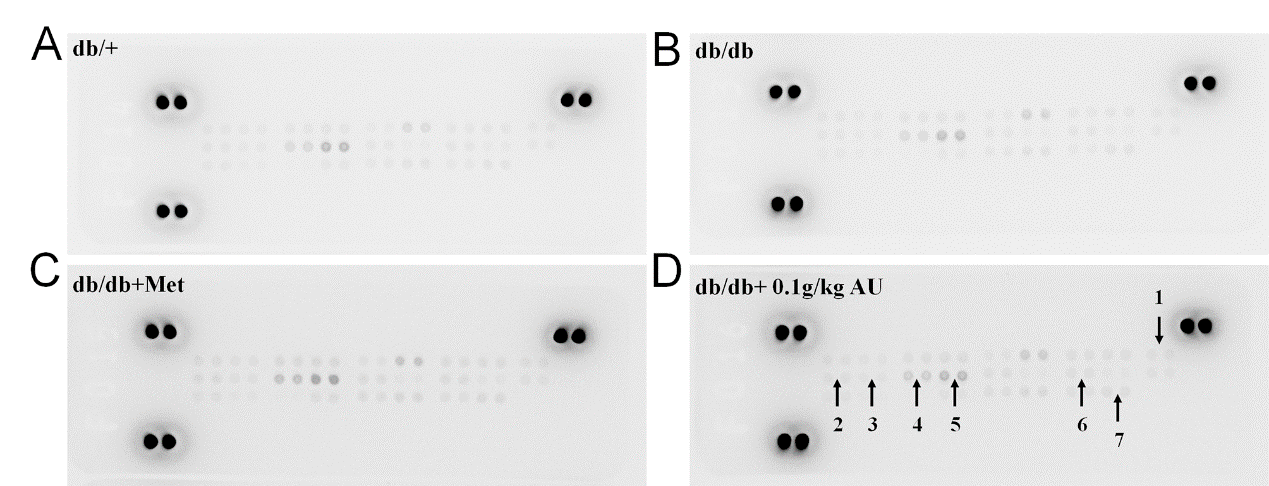
**

**Fig.S3.** The effects of Met (0.1 g/kg) and AU (0.1 g/kg) on 26 cytokines in db/db mice kidney detected by a cytokine array kit. Graphical representation of cytokine expressions of (**A**) db/+, (**B**) db/db, (**C**) Met-treated db/db and (**D**) AU-treated db/db. The arrows indicate the factors related with oxidative stress (AU group versus Met group). 1. HIF-1alpha; 2. HIF-2alpha; 3. Phospho-HSP27 (S78/S82); 4. HSP60; 5. HSP70; 6. NFkappaB1; 7. SOD2.

**
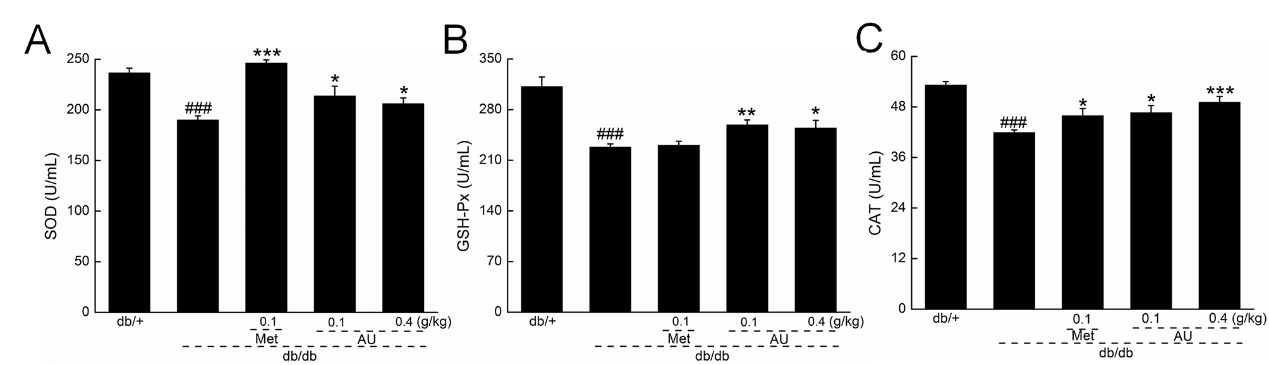
**

**Fig.S4.** The effects of AU on oxidative stress related factors in the serum of mice. Eight-week AU treatment regulated the serum level of the(**A**) SOD, (**B**) GSH-Px and (**C**) CAT in db/db mice. The data were expressed as means ± S.E.M. (n = 10) and analyzed using a one-way ANOVA. ^###^ *P* < 0.001 *versus* db/+ mice, * *P* < 0.05, ** *P* < 0.01 and *** *P* < 0.001 *versus* non-treated db/db mice. SOD: superoxide dismutase; GSH-Px: glutathione peroxidase; CAT: catalase.
